# Silencing of human T-cell leukemia virus type I gene transcription by epigenetic mechanisms

**DOI:** 10.1186/1742-4690-2-64

**Published:** 2005-10-22

**Authors:** Yuko Taniguchi, Kisato Nosaka, Jun-ichirou Yasunaga, Michiyuki Maeda, Nancy Mueller, Akihiko Okayama, Masao Matsuoka

**Affiliations:** 1 Laboratory of Virus Immunology, Institute for Virus Research, Kyoto University, Kyoto 606-8507, Japan; 2 Department of Hematology, Kumamoto University School of Medicine, Kumamoto 860-8556, Japan; 3 Laboratory of Infection and Prevention, Institute for Virus Research, Kyoto University, Kyoto 606-8507, Japan; 4 Department of Epidemiology, Harvard School of Public Health, Boston, Massachusetts 02115, USA; 5 Department of Laboratory Medicine, Faculty of Medicine, University of Miyazaki, Miyazaki 889-1692, Japan

## Abstract

**Background:**

Human T-cell leukemia virus type I (HTLV-I) causes adult T-cell leukemia (ATL) after a long latent period. Among accessory genes encoded by HTLV-I, the *tax *gene is thought to play a central role in oncogenesis. However, Tax expression is disrupted by several mechanims including genetic changes of the *tax *gene, deletion/hypermethylation of 5'-LTR. To clarify the role of epigenetic changes, we analyzed DNA methylation and histone modification in the whole HTLV-I provirus genome.

**Results:**

The *gag*, *pol *and *env *genes of HTLV-I provirus were more methylated than pX region, whereas methylation of 5'-LTR was variable and 3'-LTR was not methylated at all. In ATL cell lines, complete DNA methylation of 5'-LTR was associated with transcriptional silencing of viral genes. HTLV-I provirus was more methylated in primary ATL cells than in carrier state, indicating the association with disease progression. In seroconvertors, DNA methylation was already observed in internal sequences of provirus just after seroconversion. Taken together, it is speculated that DNA methylation first occurs in the *gag*, *pol *and *env *regions and then extends in the 5' and 3' directions *in vivo*, and when 5'-LTR becomes methylated, viral transcription is silenced. Analysis of histone modification in the HTLV-I provirus showed that the methylated provirus was associated with hypoacetylation. However, the *tax *gene transcript could not be detected in fresh ATL cells regardless of hyperacetylated histone H3 in 5'-LTR. The transcription rapidly recovered after *in vitro *culture in such ATL cells.

**Conclusion:**

These results showed that epigenetic changes of provirus facilitated ATL cells to evade host immune system by suppressing viral gene transcription. In addition, this study shows the presence of another reversible mechanism that suppresses the *tax *gene transcription without DNA methylation and hypoacetylated histone.

## Background

Human T-cell leukemia virus type I (HTLV-I) is associated with a neoplastic disease, adult T-cell leukemia (ATL), and inflammatory diseases, such as HTLV-I-associated myelopathy (HAM)/tropical spastic paraparesis (TSP) and HTLV-I-associated uveitis [1,2]. Among HTLV-I carriers, a part of infected individuals develop ATL after a long latent period. During the leukemogenesis by HTLV-I, Tax protein is considered to play a critical role through its pleiotropic actions, which include transactivation of NF-κB, CREB and SRF pathways, transrepression of *lck*, *p18 *and DNA polymerase β gene transcriptions, and functional inactivation of p53 and MAD1 [3-6]. Through these actions, Tax induces the proliferation of HTLV-I infected cells and inhibits their apoptosis, resulting in an increase in the number of infected cells. However, since Tax protein is the major target of cytotoxic T-lymphocytes (CTLs) *in vivo*, the expression also has a negative effect on the survival of ATL cells [7-9]. In some ATL cells, *tax *gene expression is inactivated by genetic and epigenetic changes, which include deletion, insertion or mutation of the *tax *gene, and DNA methylation or deletion of 5'-LTR [10-13]. Such inactivation of Tax expression is considered to allow ATL cells to escape from the host immune system.

DNA methylation of retroviruses is regarded as a host defense mechanism for inactivating retrovirus expression [14]. However, it is also recognized as a mechanism for virus-infected cells to escape from the host immune system and establish the latent state. In contrast, human immunodeficiency virus (HIV) is resistant to silencing *in vivo*. It is because HIV is frequently integrated into active transcriptional unit *in vivo *[15]. These findings coincide with the fact that HIV vigorously replicates *in vivo*. On the other hand, DNA methylation accumulated in HTLV-I 5'-LTR has been shown to silence viral gene transcription in leukemic cells [12,13]. In addition, the frequency of integration of HTLV-I provirus into transcriptional units was equivalent to that calculated based on random integration [16], which also increased the silencing. It remains unclear where and when DNA methylation occurs within the HTLV-I provirus genome.

In this study, we analyzed DNA methylation and histone modification in the whole HTLV-I provirus, and observed the progressive accumulation of DNA methylation. In addition, another reversible mechanism silenced viral gene transcription regardless of hyperacetylated promoter region.

## Results

### Analyses of DNA methylation of HTLV-I provirus

**Figure 1:**
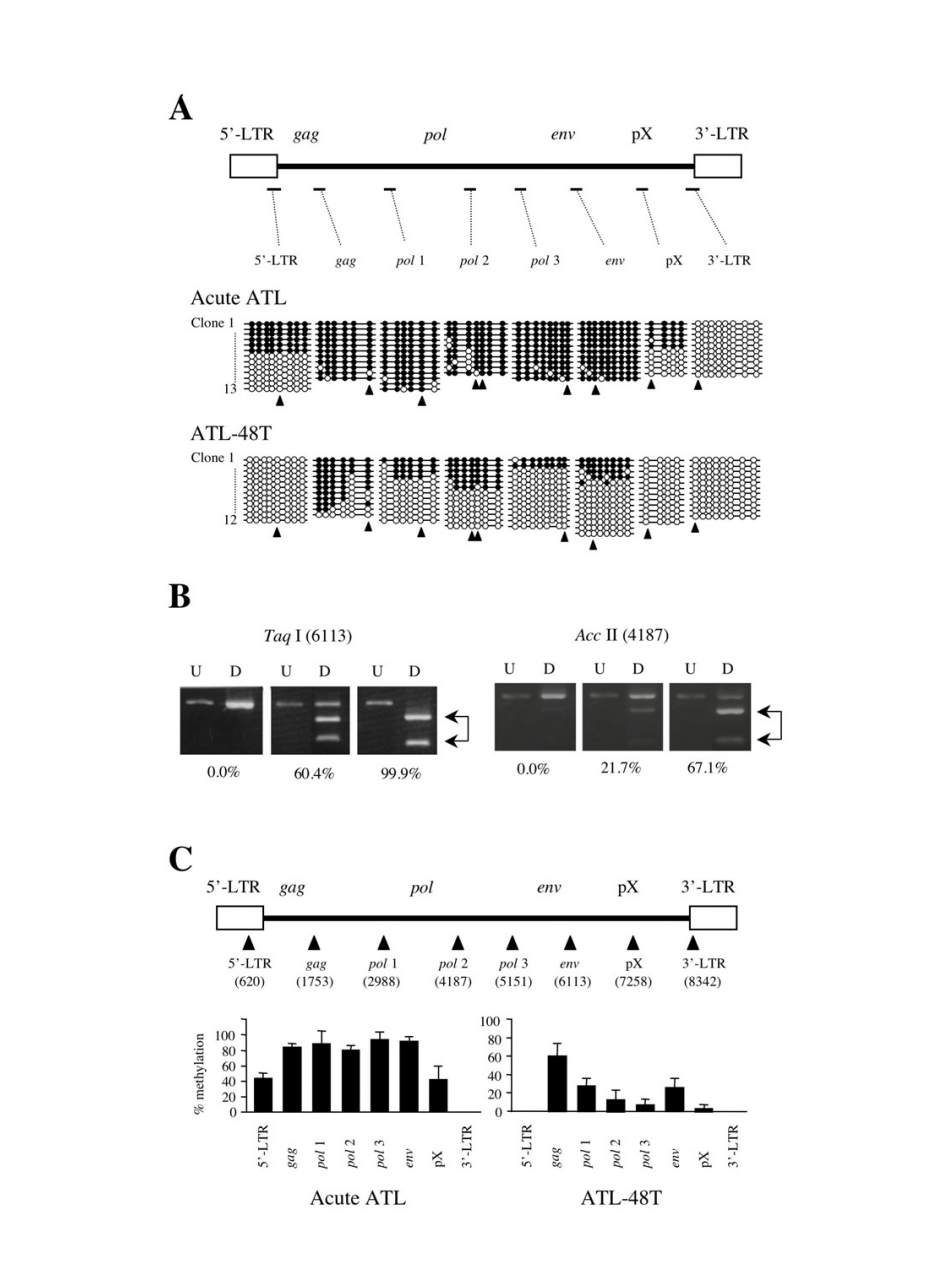
**DNA methylation of the HTLV-I provirus assessed by sodium bisulfite sequencing and COBRA. **A. DNA methylation in the HTLV-I provirus was analyzed by sodium bisulfite sequencing in a case of acute ATL and a *tax *gene-expressing cell line, ATL-48T. Eight DNA regions, which were represented as bars in A, were amplified with sodium bisulfite treated DNA. The PCR products were subcloned into plasmid DNA, and then the sequences of each clone were determined for at least ten clones of each region. Arrowheads indicate the CpG sites that were target sites for COBRA. Closed circle indicates methylated CpG, and open circle means unmethylated CpG. The number of integrated provirus has been shown in parenthesis. B. Representative data of COBRA has been shown. PCR products, which were amplified with sodium bisulfite treated DNAs, were digested with *Taq*I or *Acc*II. The extent of methylation in each CpG site was measured as described in Methods, and presented as percentages of methylated CpG. The number in parenthesis represents the position of cytidine residue in analyzed CpG site by COBRA according to Seiki et al. [41]. C. DNA methylation studied by COBRA at eight points in the provirus as shown by arrowheads. Each bar represented the extent of DNA methylation at the points shown by arrowhead. The analyses by COBRA were performed three times independently, and the extents of DNA methylation are shown by the mean ± SD. The number in parenthesis shows the position of cytidine residue of CpG site analyzed by COBRA.

To reveal DNA methylation status within the HTLV-I provirus, we analyzed the DNA methylation by sodium bisulfite sequencing and combined bisulfite restriction analysis (COBRA). Initially, DNA methylation in 5'-LTR, *gag*, *pol*, *env*, pX and 3'-LTR was identified by sodium bisulfite sequencing. In an ATL case (Fig. 1A), the internal regions of the HTLV-I provirus, including *gag*, *pol *and *env*, were heavily methylated. On the other hand, 5'-LTR and pX were partially methylated, and 3'-LTR was not methylated at all. In an ATL cell line, ATL-48T (Fig. 1A), the internal sequences of the HTLV-I provirus were partially methylated, whereas both LTRs were not methylated. Since the analyses by sodium bisulfite sequencing were time-consuming, we established the COBRA method to detect and analyze DNA methylation in a large number of samples, and then compared the results obtained with the two methods. After amplification of sodium bisulfite treated DNAs with each primer sets, the products were digested with *Taq*I or *Acc*II, which contain one (*Taq*I) or two (*Acc*II) CpG site(s) within the recognition sequences. When CpG site is methylated, the products retain CpG site, resulting in digestion by these enzymes. On the other hand, CpG is converted to UG when it is unmethylated. Therefore, PCR products are resistant to restriction enzymes (Fig. 1B). With the COBRA method, the extent of DNA methylation was quantified in eight CpG sites throughout the HTLV-I provirus: 5'-LTR (620 according to the numbering by Seiki et al. [17]), *gag *(1753), *pol *(2988, 4187 and 5151), *env *(6113), pX (7258) and 3'-LTR (8342) (Fig. 1C). The extent of DNA methylation detected by the COBRA method was well correlated with that obtained by sodium bisulfite sequencing in both cases studied, as shown in Fig. 1A and 1C.

### DNA methylation throughout the HTLV-I provirus in HTLV-l-transformed and ATL cell lines

**Figure 2:**
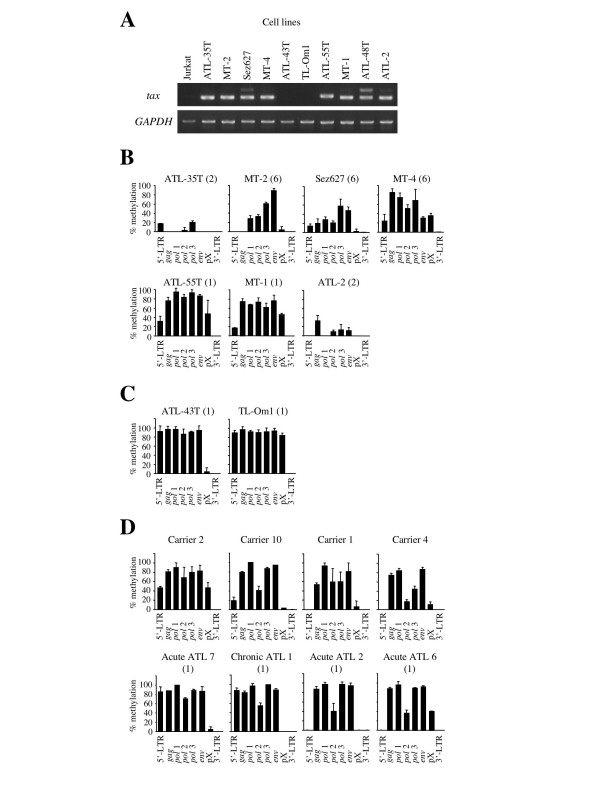
**DNA methylation in ATL cell lines, HTLV-I carriers and ATL cases. **The *tax *gene transcription in ATL cell lines was studied by RT-PCR (A), and the expression of *GAPDH *gene has been used as a control. DNA methylation throughout the HTLV-I provirus was studied by COBRA in *tax *gene-expressing (B) and non-expressing cell lines (C). Furthermore, DNA methylation was also analyzed in 20 carriers and 20 ATL cases by COBRA, and representative patterns of DNA methylation are shown in D. The number of HTLV-I provirus has been analyzed by Southern blot method, and shown in the parenthesis (B, C and D). Each bar indicates the extent of DNA methylation that was calculated by COBRA.

Using the COBRA method, we analyzed the DNA methylation throughout the whole HTLV-I provirus of the cell lines (Fig. 2B and 2C). In addition, we also analyzed the *tax *gene transcription by RT-PCR (Fig. 2A) and the number of integrated HTLV-I proviruses in each cell lines by Southern blot method. Among the *tax *gene-expressing cell lines (ATL-35T, MT-2, Sez627, MT-4, ATL-55T, MT-1 ATL-48T and ATL-2) (Fig. 2A), internal sequences from *gag *to pX were variably methylated. However, 5'-LTR was not methylated or partially methylated, while 3'-LTR was not methylated in all cell lines (Fig. 2B). In ATL-43T and TL-Oml, which did not show *tax *gene transcription (Fig. 2A), 5'-LTR and the internal sequences were heavily methylated (Fig. 2C), indicating the close correlation between the extents of DNA methylation of the provirus, particularly 5'-LTR, and *tax *gene transcription. As previously reported, the treatment by 5-aza-deoxy-cytidine can recover the *tax *gene expression of these cell lines, indicating that the latent state by DNA methylation of 5'-LTR is reversible [13].

Among cell lines, HTLV-I provirus tends to be not so methylated in cell lines with higher copy number of provirus (Fig. 2). The finding that cell lines with higher integrated provirus number contain hypomethylated provirus is speculated to reflect the higher transcription of viral genes.

### DNA methylation of the HTLV-I provirus in ATL and HTLV-I carrier states

**Figure 3:**
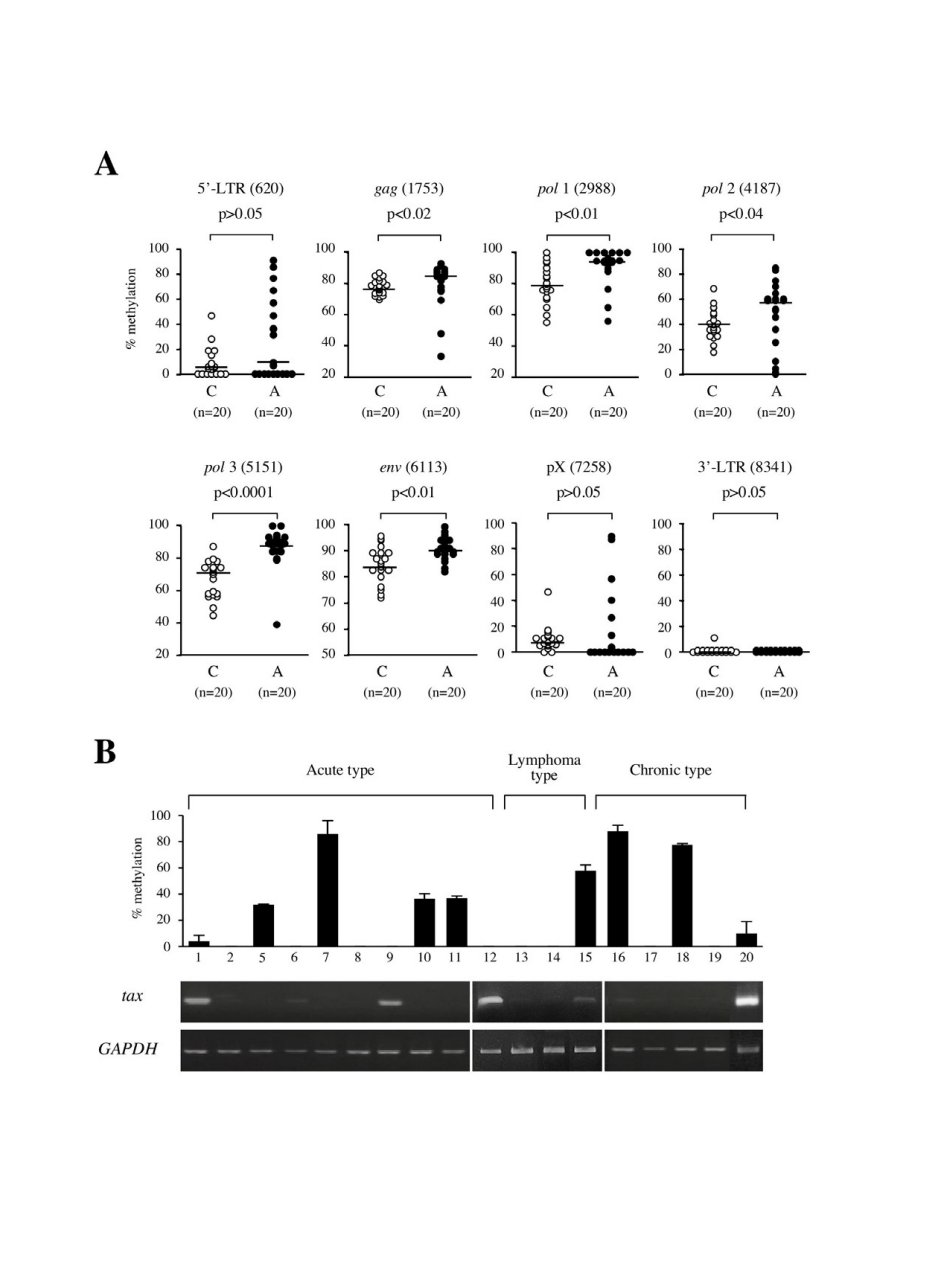
**Comparison of the DNA methylation in carriers and ATL cases. **A. DNA methylation at eight different regions in the HTLV-I provirus was compared between carriers (C) and ATL cases (A). DNA methylation was quantified by COBRA in 20 carriers and 20 ATL cases. Each sample was analyzed three times by COBRA at each site, and circles indicate mean values of DNA methylation. The differences of DNA methylation are statistically significant in the *gag*, *pol *and *env *regions by the Mann-Whitney's U-test. Horizontal bars represent median of DNA methylation in each group. B. The relation between *tax *gene transcription and DNA methylation of 5'-LTR in the fresh ATL cells has been shown. DNA methylation of 5'-LTR was quantified by COBRA assay and the *tax *gene transcripts were detected by RT-PCR.

Next, we analyzed the DNA methylation of the whole HTLV-I provirus in ATL patients and HTLV-I carriers. Although 5'-LTR is frequently deleted in ATL cells [10], we omitted such ATL cases lacking 5'-LTR in this study. In Fig. 2D, we showed the representative pattern of DNA methylation of whole HTLV-I provirus in carriers and ATL patients. In ATL samples, the *gag*, *pol *and *env *regions were heavily methylated, whereas 5'-LTR was not methylated or partially methylated (Fig. 2D and 3A). On the other hand, 5'-LTR was scarcely methylated and the *gag*, *pol *and *env *regions seemed to be less methylated in HTLV-I carriers (Fig. 2D and 3A). We compared DNA methylation of these different eight regions between 20 carriers and 20 ATL cases (Fig. 3A). These differences in DNA methylation were statistically significant in the *gag*, *pol *and *env *regions between the ATL cases and HTLV-I carriers by the Mann-Whitney's U-test. These data suggested that DNA methylation initially occurred in the *gag*, *pol*, and *env *regions, and that DNA methylation of the provirus accumulated during disease progression from the carrier state to the leukemic stage. The frequency of DNA methylation of 5'-LTR did not differ between carriers and ATL patients. However, the extent of DNA methylation among methylation-positive cases was higher in ATL cases than in carriers (p = 0.001). Among ATL cases, the relationship between DNA methylation of 5'-LTR and *tax *gene transcription was analyzed (Fig. 3B), and the transcript was detected in six cases. In four cases with relative higher amount of *tax *gene transcripts (Case 1, 9, 12, 20), 5'-LTR was not methylated or slightly methylated. This finding suggests that higher expression of *tax *gene is associated with unmethyalted or slightly methylated 5'LTR, however, other mechanism(s) silences the *tax *gene transcription in ATL cells. There is no statistical correlation between the *tax *gene transcription and DNA methylation of 5'-LTR

### DNA methylation of HTLV-I provirus after seroconversion

**Figure 4:**
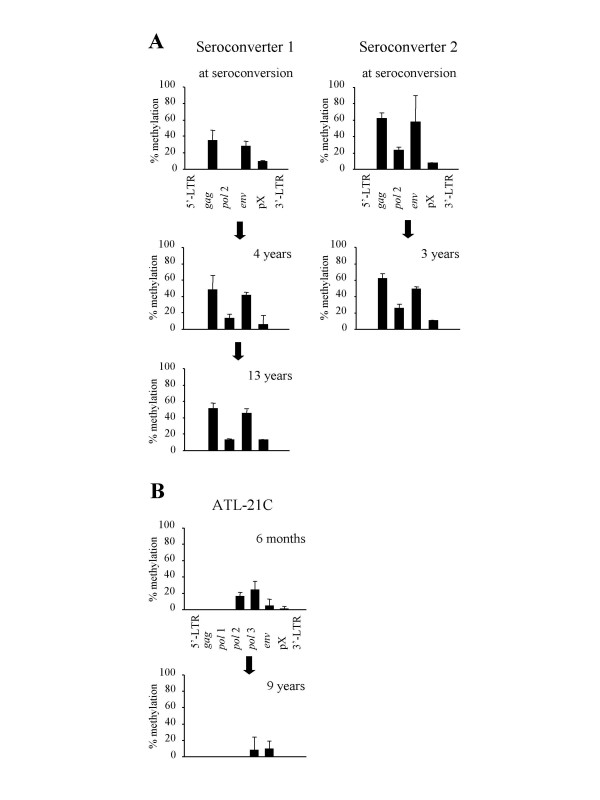
**Sequential analyses of the DNA methylation in seroconverters and a cell line. **DNA methylation was analyzed by COBRA in sequential samples from seroconverters (A) and in a cell line, ATL-21C, (B) cultured *in vitro *for more than 9 years. DNA methylation was analysed by COBRA three times, and each bar indicates mean ± SD.

The analyses of DNA methylation suggest that it first occurs around the *gag*, *pol *and *env *regions, and then progresses in both the 5' and 3' regions. To study the changes in DNA methylation after infection, we analyzed sequential DNA samples from seroconverters. As shown in Fig. 4A, DNA methylation already existed in the *gag*, *pol *and *env *regions at the seroconversion. In seroconverter 1, DNA methylation was slightly increased at 4 and 13 years after the seroconversion. Increase of DNA methylation at *pol *region (4187) is statistically significant 13 years later in seroconverter 1 (p = 0.02, by a Student's t-test). On the other hand, there was little change in the DNA methylation in seroconverter 2, although the HTLV-I provirus was already heavily methylated at the seroconversion. When DNA methylation of seroconverters was compared with that in carriers (Fig. 3A), provirus of carriers was more methylated in carriers than that of seroconverters (p < 0.01 by a Student's t-test) except for *pol*2 in seroconverter 2, and pX region. It suggests that DNA methylation of provirus accumulates during a latent period after seroconversion.

We established an HTLV-I-transformed cell line, ATL-21C, and cultured for over 9 years *in vitro*, and analyzed the DNA methylation of the HTLV-I provirus. Slight DNA methylation was detected in the *pol*, *env *and pX regions at 6 months after culture, however, it did not increase after 9 years. This indicates that the DNA methylation of HTLV-I provirus did not change after long-term *in vitro *culture (Fig. 4B). On the other hand, the *p16 *gene in this cell line was not methylated at 6 months after culture, but heavily methylated after 9 years (data not shown). A comparison with the data from the seroconverters suggests that DNA methylation of the HTLV-I provirus tends to accumulate *in vivo.*

### Association with DNA methylation in the neighboring host genome

**Figure 5:**
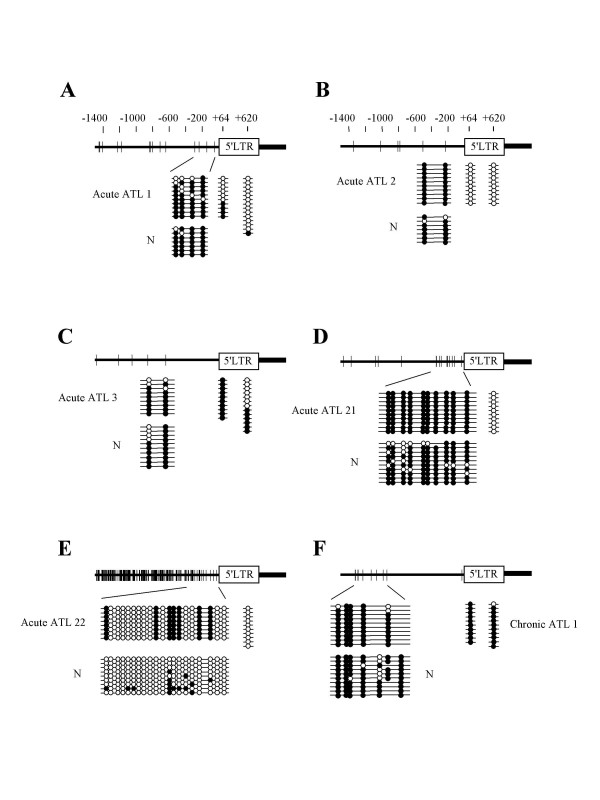
**DNA methylation of provirus is not associated with methylated CpG sites in the genome. **Integration sites of HTLV-I provirus in leukemic cells have been determined by inverse PCR, and then DNA methylation in genome has been analyzed by sodium bisulfite sequencing. DNA methylation of 5'-LTR was also analyzed by sodium bisulfite sequencing method. Vertical bars represent CpG sites. Open circle indicates unmethylated CpG site, and closed one means methylated CpG site. N: normal PBMCs from non-carrier donor.

It is possible that the HTLV-I provirus integrated into the heterochromatin or hypermethylated regions tends to be silenced [18], and that such HTLV-I-infected cells are selected *in vivo*. Therefore, we analyzed the DNA methylation of the host genome around the integration sites of the HTLV-I provirus. We first determined the integration sites of the HTLV-I provirus in ATL cells, and then analyzed the DNA methylation of genomic DNAs around the integration sites in both ATL cells and normal PBMCs from a non-carrier donor. When genomic DNAs neighboring integration sites were heavily methylated (Fig. 5), 5'-LTR was not methylated in three cases (acute ATL 1, 2 and 21) while they were methylated in two cases (acute ATL 3 and chronic ATL 1). In acute ATL 22, both genomic DNA and 5'-LTR were not so methylated. Thus, DNA methylation in the neighboring genomic regions was not correlated with the methylation status of the provirus among these cases.

### Histone modification of the HTLV-I provirus

**Figure 6:**
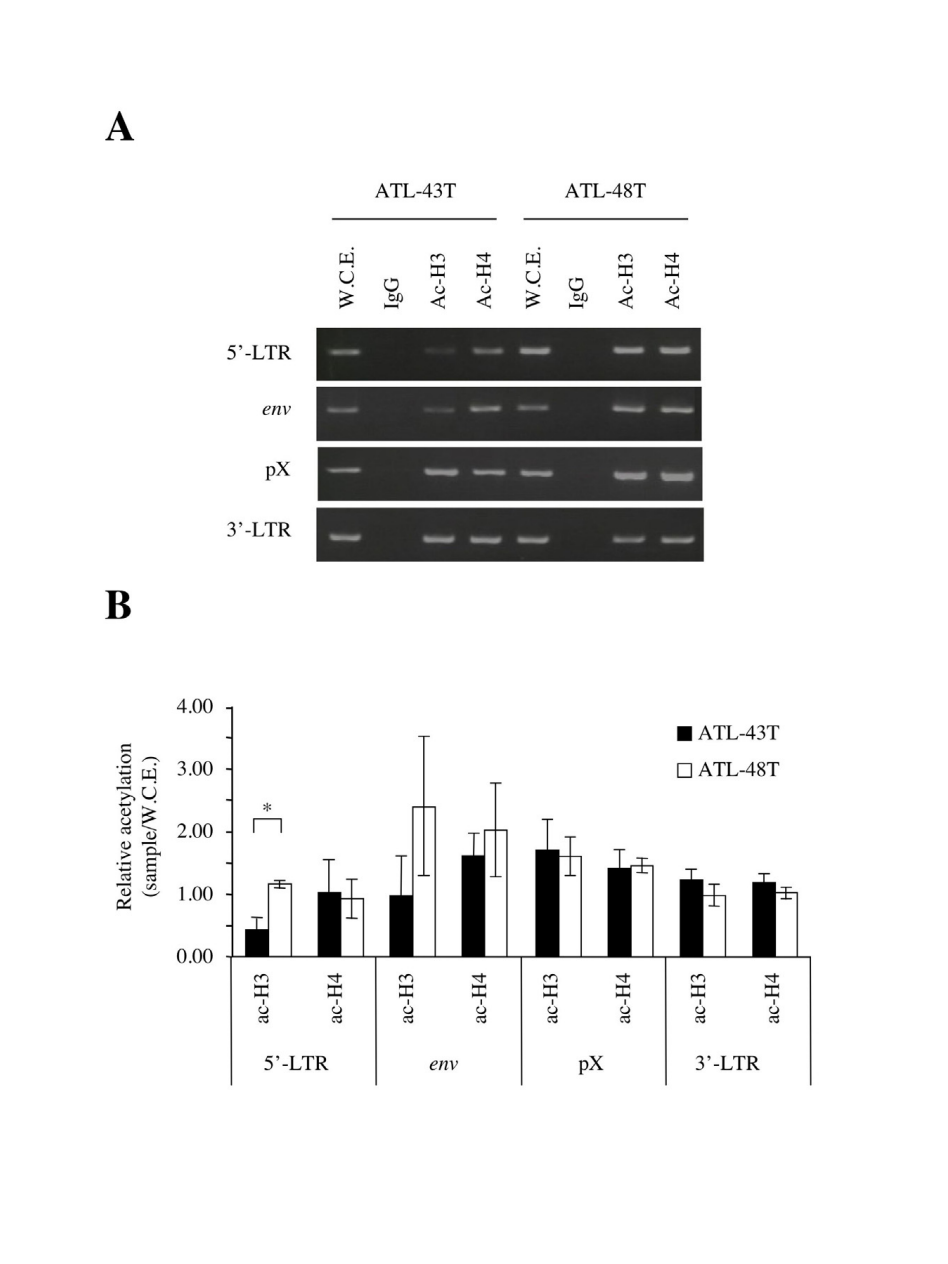
**Histone modifications in ATL cell lines. **Acetylation of histone was analyzed in *tax *gene-expressing (ATL-48T) and non-expressing (ATL-43T) cell lines by ChIP assays with anti-acetyl-Histone H3 or H4 (A and B) at four different regions (for 5'-LTR, *env*, pX and 3'-LTR) of the provirus. Representative data has been shown in *A*. W.C.E.: whole cell extract. ChIP assay was performed three times and quantified as described in Methods. Values are means ± SD(B). *:p < 0.002.

It has been demonstrated that DNA methylation of 5'-LTR is associated with histone deacetylation and silencing of viral gene transcription in cell lines [13]. When ATL-43T, in which *tax *gene transcription was silenced by hypermethylation of 5'-LTR, was compared with a *tax *gene-expressing cell line, ATL-48T, a difference was found in the acetylation of histone H3 in 5'-LTR (Fig. 6A and 6B). The histone H3 of 5'-LTR was hypoacetylated in ATL-43T compared with ATL-48T, whereas there were no differences in pX or 3'-LTR among these cell lines. Since the number of HTLV-I provirus in ATL-43T and -48T is one and two copies respectively, and acetylation of histone H3 in pX and 3'-LTR was similar in both cell lines, the number of provirus was thought to have no influence on the results of ChIP assay in 5'-LTR.

**Figure 7:**
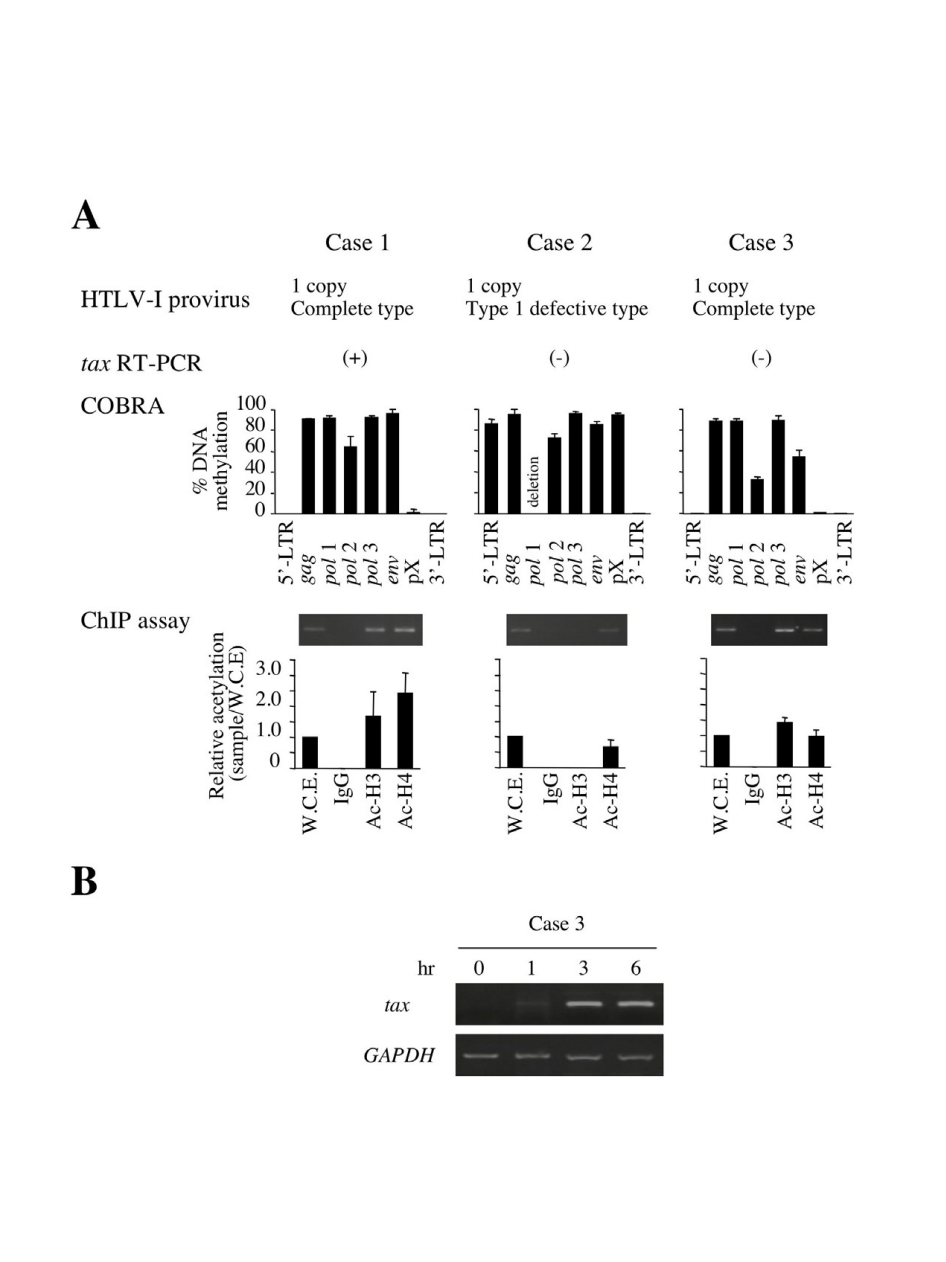
**DNA methylation and histone modifications in fresh ATL cases. **A. The relationships among DNA methylation, *tax *gene expression and histone modification in 5'-LTR were analyzed in three ATL cases. Cases 1 and 3 have one copy of the complete HTLV-I provirus, while Case 2 has a defective provirus that lacks part of the *pol *gene. DNA methylation was analyzed by COBRA. The *tax *gene transcripts could be detected in Case 1, but not in Cases 2 or 3, by RT-PCR. ChIP assays were also performed using primers for 5'-LTR to analyze acetylation of histone H3 (Ac-H3) and H4 (Ac-H4). W.C.E.: whole cell extract. B. Recovery of *tax *gene expression *ex vivo*. The PBMCs isolated from Case 3 were immediately cultured *ex vivo *for several hours and tested the transcription of *tax *mRNA by RT-PCR.

However, the *tax *gene transcription is silenced in about 20% of ATL cases despite no or partial methylation of 5'-LTR (Fig. 3B) [13], suggesting that there is aother mechanism(s) for suppressing viral gene transcription. To address this question, we studied the histone modification of 5'-LTR in fresh ATL cells with or without *tax *gene transcription. In a case with *tax *gene expression, 5'-LTR was not methylated and histone H3 was hyperacetylated (Fig. 7A, Case 1). On the other hand, in Case 2 with heavily methylated 5'-LTR, histone H3 was hypoacetylated in 5'-LTR, which was consistent with the lack of detection of *tax *gene transcription in this case. However, in Case 3, *tax *gene transcription could not be detected regardless of 5'-LTR hyperacetylation. After *in vitro *culture, such cells showed *tax *gene transcription within one hour (Fig. 7B). Although both Cases 1 and 3 exhibited hyperacetylation of 5'-LTR, *tax *gene transcription was silenced in Case 3.

## Discussion

DNA methylation is regarded as a host defense mechanism for inactivating transportable elements such as retroviruses to inhibit viral transcription and the generation of new viruses. On the other hand, it also renders the provirus into a latent state, resulting in the establishment of latent infection. However, it remained unclear how and when the provirus was methylated, and whether DNA methylation changed *in vivo.*

Tax has the remarkable potency to promote the proliferation of infected cells [3], however, it is also a major target of CTL *in vivo *[8]. Therefore, HTLV-I controls *tax *gene expression by own viral proteins, Rex [19], p30 [20,21] and HBZ [22]. In the leukemic cells, several mechanisms have been identified to suppress or abolish Tax expression, including genetic changes of *tax *gene, deletion of 5'-LTR, and DNA methylation of 5'-LTR. In this study, DNA methylation was shown to occur in internal provirus sequences, such as the *gag*, *pol *and *env *regions, and then extend to 5' (5'-LTR) and 3' (pX) regions. Since DNA methylation of 5'-LTR is associated with *tax *gene transcription, the finding that 5'-LTR was more highly methylated in ATL cells than in carriers, among cases with methylated 5'-LTR, suggests that such HTLV-I-infected cells and ATL cells with the methylated provirus, which produce lower amounts of viral proteins, are selected *in vivo *by the host immune system. In this regard, HTLV-I is quite different from another human retrovirus, HIV-1. HIV-1 vectors were resistant to gene silencing *in vivo *[23,24]. It is noteworthy that the number of CpG sites in the U3 region of HIV-1 LTR (9 sites in LTR of NL43) is much fewer than that of HTLV-I (47 sites in LTR of ATK). This is consistent to the previous report that transcriptional suppression was not associated with DNA methylation of HIV-1 provirus [25]. In addition, HIV-1 provirus is frequently integrated within transcriptional units, which encode the genes that are transcribed in T-cells [15,26]. In such regions, it is possible that HIV-1 tends to escape from transcriptional silencing that is observed in the heterochromatin region such as alphoid repetitive sequences [18]. These data suggest that HIV-1 is more resistant to gene silencing than HTLV-I. Alternatively, it is possible that HTLV-I takes advantage of susceptibility to DNA methylation to escape from the host immune system.

This study shows that 3'-LTR is unmethylated in carriers and ATL cells while 5'-LTR is methylated in about half of cases. In HTLV-I, *HTLV-I bZIP *(*HBZ*) *gene *is encoded by minus strand of provirus [22,27]. We observed that *HBZ *gene was transcribed in all ATL cells, suggesting that *HBZ *gene play a critical role in growth of HTLV-I infected cells and ATL cells (submitted for publication). The finding that 3'-LTR is unmethylated in all ATL cases and carriers suggests that *HBZ *gene transcription is important for proliferation of ATL and HTLV-I infected cells.

Why does DNA methylation occur from the internal sequences of the HTLV-I provirus? Since CpG island is recognized as DNA region that is susceptible to DNA methylation, we analyzed HTLV-I provirus by the criterion by Takai and Jones [28]. CpG islands are present throughout the provirus in 5'-LTR-*gag *(1–1360), *pol *(3876–4509), *env *(5648–6166), *env*-pX (6446–7561), and pX-3'-LTR (8212–9045) regions. Therefore, the presence of CpG island could not explain why DNA methylation occurred in the internal region of HTLV-I provirus. Among tumor-suppressor genes, which are transcriptionally silenced by DNA methylation, the exon regions are first methylated, and then DNA methylation progresses to the promoter region [29]. When the promoter region is heavily methylated, the transcription of the corresponding gene is silenced. Since 5'-LTR is the promoter/enhancer for viral gene transcription, there might be a similar scenario between the exon/promoter and DNA methylation in both virus and tumor-suppressor genes. Thus, it is possible that gene coding regions are first methylated and DNA methylation spreads to the promoter region of provirus, 5'-LTR.

Transcriptional silencing of *tax *gene in spite of hyperacetylated histone H3 is recognized as another mechanism to suppress the viral gene transcription in addition to DNA methylation. The prompt recovery of *tax *gene expression after *in vitro *culture suggests the presence of an inhibitory factor(s) that binds to 5'-LTR, and suppresses the viral gene transcription *in vivo*. It is noteworthy that this phenotype is very similar to that of a mouse T-cell line transfected with an HTLV-I LTR-derived reporter plasmid [30]. In that study, a green fluorescent protein-fused Tax (*Gax*) gene was transfected into a mouse T-cell line, EL-4, and the transduced cells were then injected into Tax-immunized and non-immunized mice. Although Tax-induced cytotoxic T-cells suppressed the expression of the *Gax *gene *in vivo*, its expression was shown to recover within three hours when the transduced cells were transferred to *in vitro *culture. This phenotype resembles that observed in Case 3 in Fig. 7. Considering that Tax is the major target of CTL *in vivo*, and at the same time, confers growth advantages on the infected cells, such reversible suppression of *tax *gene expression is thought to be suitable for the survival of HTLV-I infected cells, and ATL cells. In this regard, potentiation of anti-Tax immunity might protect against the development of ATL when combined with possible therapeutics to induce Tax expression [31]. For this purpose, the mechanism for silencing viral transcription regardless of histone H3 hyperacetylation should be studied.

In general, gene silencing is associated with several different mechanisms. DNA methylation in the promoter region silences the gene transcription, whereas gene silencing is often not associated with DNA methylation [32,33]. In such situations, methylation of H3K9 is linked with loss of transcriptions [34]. It is possible that silencing of viral gene transcription renders proviral DNA vulnerable to methylation. Once proviral DNA is methylated, such silencing would be fixed unless such cells are treated with demethylating agents such as 5-aza-deoxy-cytidine.

DNA methylation of the HTLV-I provirus did not accumulate in a cell line that was cultured *in vitro *for more than 9 years. The finding that the *p16 *gene was heavily methylated in this cell line excluded the possibility that hypermethylation did not occur in this cell line due to aberrant methylation machinery. Among the seroconverters, the provirus was heavily methylated in internal regions such as *gag*, *pol *and *env*. Taken together, DNA methylation in the provirus is considered to reflect the selection *in vivo*. Since the growth of *in vitro *HTLV-I-transformed cell lines depends on Tax expression, cells with suppressed expression of the *tax *gene do not have the growth advantage *in vitro*. However, the immune system exerts selection of the infected cells with suppressed *tax *gene expression *in vivo.*

Recently, both 5'- and 3'-LTR have been reported to be transcriptionally active, and transcriptional factors and Tax bind equally to both [35]. 3'-LTR may activate the transcription of cellular genes, which are located in the downstream of integration sites. In addition, unmethylated 3'-LTR is critical for transcription of the *HBZ *gene. Since 5'-LTR is a promoter/enhancer for viral gene transcription, selective methylation of 5'-LTR is considered to silence the transcription of viral genes.

## Conclusion

We have demonstrated how DNA methylation of HTLV-I provirus occurred, and how it suppressed viral gene transcription. When 5'-LTR was heavily methylated, viral transcription was silenced, which is thought to reflect the immune system selection *in vivo*. In addition, mechanisms other than DNA methylation suppresses viral gene transcription regardless of histone H3 hyperacetylation. The mechanism of such suppression requires further investigation.

## Methods

### Cells

HTLV-I-associated cell lines (MT-1, MT-2, MT-4, ATL-2, TL-Oml and Sez627) were cultured in RPMI1640 medium supplemented with 10% fetal bovine serum and penicillin/streptomycin. For interleukin-2-dependent cell lines (ATL-43T, 48T and 55T), 100 U/ml of recombinant interleukin-2 (Shionogi, Osaka) was added to the medium. Peripheral blood mononuclear cells (PBMC) or lymph node cells were isolated from HTLV-I carriers and ATL patients after informed consent was obtained. The polyclonal integration of HTLV-I provirus in carriers has been shown by inverse PCR [36], and provirus load was determined by real-time PCR as reported previously [37].

### Sodium bisulfite treatment of genomic DNA

Sodium bisulfite treatment was performed as described previously [29]. Briefly, 1–3 μg of genomic DNA was denatured in 0.3 N NaOH at 37°C for 15 min, and 1 μg of salmon sperm DNA was added to each sample as a carrier. Sodium bisulfite (pH 5.0) and hydroquinone were added to each sample to final concentrations of 3 M and 0.05 mM, respectively. The reaction was performed at 55°C for 16 h and the samples were then desalted using the Wizard DNA Clean-Up System (Promega, Madison, WI). Finally, samples were desulfonated in 0.3 N NaOH at 37°C for 15 min.

### Sequencing of sodium bisulfite-treated genomic DNA

The sodium bisulfite-treated DNA (200–500 ng) was used as a template for PCR amplification of eight HTLV-I provirus regions. The PCR reactions were performed using FastStart Taq DNA Polymerase (Roche, Mannheim, Germany). The PCR primer pairs and annealing temperatures are shown in Table 1. The amplified PCR products were purified and subcloned into pGEM-T Easy vectors (Promega). For each region, at least 10 clones were sequenced using Big Dye Terminator v3.1 Cycle Sequencing Kit (Applied BioSystems, Foster City, CA) and ABI3100 autosequencer (Applied Biosystems).

**Table 1:** Primer sets for COBRA and ChIP assay

	Site in HTLV-I^a^		Forward primer	Reverse primer	Anneal (°C)	Enzyme for COBRA
COBRA	620	1st	5'-TTTGGAGTTTATTTAGATTTAG-3'	5'-CCAATAATAAACRACCAACCC-3'	45	*Taq*I
	(5'-LTR)	2nd	5'-GTTTTGTTTGATTTTGTTTGT-3'	5'-AAAAAAATTTAACCCATTACC-3'	49	
	1753	1st	5'-GGGAGTGTTAAAGATTTTTTTTGGG-3'	5'-ACTCCAATAACCTACTTTCCC-3'	55	*Taq*I
	(*gag*)	2nd	5'-TTTATTTTTTAAGGTTTGGAGGAG-3'	5'-TTAAAAATCCAAATCTAACAAACCC-3'	55	
	2988	1st	5'-GTTAAAAAGGTTAATGGAATTTGG-3'	5'-CCTCTAAAAATAATAATAAATCCTC-3'	52	*Taq*I
	(*pol*)	2nd	5'-GGGTTTTTTGATTTGTTTAGTTTG-3'	5'-AAACTTACTAAAAAAATATCATCC-3'	51	
	4187	1st	5'-GGGTGAAATTGTGTAGTTTTGTAGG-3'	5'-CCTATTTTCAAACGAATCTACCTCC-3'	57	*Acc*II
	(*pol*)	2nd	5'-GTGATTAGTAGGGTATTTGTGAGAG-3'	5'-ATTATCACAAAAATCATTCCCCC-3'	52	
	5151	1st	5'-GGTATTATTTTAAGTTTTTTGG-3'	5'-CTCCAATTATAAAAATACAACAAC-3'	46	*Taq*I
	(*pol*)	2nd	5'-GTTAGTGGAAAGGATTATAGGAGG-3'	5'-AACTTACCCATAATATTAAAAATC-3'	51	
	6113	1st	5'-GGATTTATTGTTTTGATTTTTAG-3'	5'-CTTTACATAATCCTCCTTACTCCC-3'	51	*Taq*I
	(*env*)	2nd	5'-GGATTTATTGTTTTGATTTTTAG-3'	5'-CCCAAAACAAAAAATCAAAACC-3'	53	
	7258	1st	5'-GAGGTGGYGTTTTTTTTTTTGG-3'	5'-CCTTAAAAATCTTAAAAATTCTC-3'	47	*Taq*I
	(pX)	2nd	5'-AAGGATAGTAAATYGTTAAGTATAG-3'	5'-CCCAAATAATCTAATACTCTAAAC-3'	50	
	8342	1st	5'-YGATGGTAYGTTTATGATTTTYGGG-3'	5'-ACCCCCTCCTAAACTATCTCC-3'	57	*Taq*I
	(3'-LTR)	2nd	5'-YGATGGTAYGTTTATGATTTTYGGG-3'	5'-AACTCCTACTAATTTATTAAACC-3'	52	
	5'-LTR^b^		5'-GCTTTGCCTGACCCTGCTTGC-3'	5'-AAGATTTGGCCCATTGCCTAGGG-3'	63	
	*env*		5'-TGCCAGCCTCTCCACTTGGCACG-3'	5'-ATGGAGCCGGTAATCCCGCCAGC-3'	64	
	pX		5'-AAGGATAGCAAACCGTCAAGCACAG-3'	5'-CCCAGGTGATCTGATGCTCTGGAC-3'	63	
	3'-LTR		5'-CCCCTCATTTCTACTCTCACACGGC-3'	5'-TGGGTGGTTCTTGGTGGCTTCCC-3'	64	

^a ^Nucleotide position corresponding to that of ATK. This number means the cytidine of CpG sites analysed.

^b^For ChIP assay, we used primers to amplify the indicated regions.

### Combined bisulfite restriction analysis (COBRA)

For COBRA, eight different regions of HTLV-I provirus were amplified with sodium bisulfite treated genomic DNAs using each primer sets as shown in Table 1. The nested PCR reactions were performed using FastStart Taq DNA Polymerase (Roche) with the following condition: 5 minutes at 95°C for denaturation, 40 cycles of 30 sec at 95°C, 30 sec at each annealing temperature (Table 1), 30 sec at 72°C, and 2 min at 72°C for final extension. The PCR products were digested for at least 4 hrs with an appropriate restriction enzyme (*Taq*I or *Acc*II) that had a single recognition site within each product [38]. When CpG site within amplified region was methylated, it was resistant to sodium bisulfite treatment, resulting in digestion by these enzymes. On the other hand, since unmethylated CpG was converted to UG by sodium bisulfite treatment, these enzymes could not digest the amplified DNAs. The digested PCR products were separated in a 3% Nusieve 3:1 agarose (BMA, Rockland, ME) gel. The intensity of each fragment was determined using ATTO Densitograph Ver. 4.0 (ATTO, Tokyo, Japan), and the extent of DNA methylation was calculated as follows: % methylation = 100 × (digested PCR products/undigested+ digested PCR products).

### Southern blot analyses

To determine the number of integrated HTLV-I provirus, we performed Southern blot method using HTLV-I probe as described previously [10]. In brief, 5 μg of DNA were digested with *Eco*RI, separated by electrophoresis in a 0.7% agarose gel, and transferred to nylon membrane (Hybond N+, Amersham Biosciences, Piscataway, NJ). The membrane was hybridized to the alkaline phospatase labeled pX probes. 0.9 kb PCR product of HTLV-I pX region derived from HTLV-I clone λ23-3 was used as probe [39]. DNA probe was labeled, and hybridized to the membrane with Gene Images AlkPhos Direct Labelling and Detection system (Amersham Biosciences).

### Inverse-long PCR

To check the HTLV-I integration in PBMCs of carriers, we analyzed the genomic DNAs from carriers by inverse-long PCR method as described previously [36]. In brief, genomic DNA was digested with *Eco*RI, and then ligated with T4 DNA ligase. Circularized DNA was digested with *Mlu*I that cut the provirus at pX region to prevent amplification of provirus itself. Then, treated genomic DNA was amplified with primers as follows: Long-IPCR-F: 5'-TGCCTGACCCTGCTTGCTCAACTCTACGTCTTTG-3', Long-IPCR-R 5'-AGTCTGGGCCCTGACCTTTTCAGACTTCTGTTTC-3'. PCR condition was as follows: 2 min at 98°C for denaturation, 5 cycles (30 sec at 98°C, 10 min at 64°C), followed by 35cycles (30 sec at 94°C, 10 min at 64°C) and 15 min at 72°C for final extension. The PCR products were subcloned into plasmid DNA and their sequences were determined.

### DNA methylation in neighboring regions of HTLV-I integration sites

The integration sites of HTLV-I provirus has been determined by inverse long PCR, and DNA methylation of genomic DNAs neighboring integration sites was determined in both ATL cells and PBMCs. The nested PCR reactions were performed using FastStart Taq DNA Polymerase (Roche) with the following condition: 5 minutes at 95°C for denaturation, 40 cycles of 30 sec at 95°C, 30 sec at each annealing temperature (Table 2), 30 sec at 72°C, and 2 min at 72°C for final extension.

**Table 2:** Primer sets and annealing temperatures for genome specific PCR

	Case	Locus		Forward primer	Reverse primer	Anneal (°C)
Primers for case	Acute ATL 1	5q11.1	1st	5'-TTTGGAGAGGGAATTTTATATTG-3'	5'-ACCCCCTCCTAAACTATCTCC-3'	55
			2nd	5'-GGAGTGTAGAGATGTAGTTTTGG-3'	5'-ACCCCCTCCTAAACTATCTCC-3'	50
	Acute ATL 2	8p23.1	1st	5'-GAGAAATTTGTGTTGATTTTATTAG-3'	5'-ACCCCCTCCTAAACTATCTCC-3'	47
			2nd	5'-TTAGTGGTAGATTAAGTTAAAG-3'	5'-ACCCCCTCCTAAACTATCTCC-3'	45
	Acute ATL 3	1q31.1	1st	5'-GGTAGAAATTATAGGTTTTTGTAGG-3'	5'-ACCCCCTCCTAAACTATCTCC-3'	51
			2nd	5'-GTTATTTGTGAAGTAAGATGTTTTG-3'	5'-ACCCCCTCCTAAACTATCTCC-3'	53
	Acute ATL 21	15q24.3	1st	5'-GAGGTGGATTTTTATTTTATTG-3'	5'-ACCCCCTCCTAAACTATCTCC-3'	52
			2nd	5'-GGTTTTTGATTATATTTGGGGAG-3'	5'-ACCCCCTCCTAAACTATCTCC-3'	54
	Acute ATL 22	19q13.11	1st	5'-GTTAGTTGTTAGAGAGTTTTTTGG-3'	5'-ACCCCCTCCTAAACTATCTCC-3'	52
			2nd	5'-AAGATTATTTAGTTTTTTGGGG-3'	5'-ACCCCCTCCTAAACTATCTCC-3'	54
	Chronic ATL 1	1p22.1	1st	5'-GGGTTTGAAGTTTTTTTTGTAGG-3'	5'-ACCCCCTCCTAAACTATCTCC-3'	53
			2nd	5'-AAGATTATTTAGTTTTTTGGGG-3'	5'-ACCCCCTCCTAAACTATCTCC-3' (5'-LTR U3)	50
Primers for human genome		5q11.1	1st	5'-TTTGGAGAGGGAATTTTATATTG-3'	5'-CCCAAACTAATCTTCAACTCC-3'	52
			2nd	5'-GGAGTGTAGAGATGTAGTTTTGG-3'	5'-CCACCATAAAAAACCCTCCC-3'	54
		8p23.1	1st	5'-GAGAAATTTGTGTTGATTTTATTAG-3'	5'-AATATCACTATAACAATAACCAC-3'	46
			2nd	5'-TTAGTGGTAGATTAAGTTAAAG-3'	5'-CTCTCAACAAATTCCATCTTTCC-3'	49
		1q31.1	1st	5'-GGTAGAAATTATAGGTTTTTGTAGG-3'	5'-CACCATTAAACAAACTAAATTCTC-3'	51
			2nd	5'-GTTATTTGTGAAGTAAGATGTTTTG-3'	5'-CACATAAAAAAACCCACACAATC-3'	53
		15q24.3	1st	5'-GAGGTGGATTTTTATTTTATTG-3'	5'-ATCTACCTAAAAAACCCACCC-3'	52
			2nd	5'-GGTTTTTGATTATATTTGGGGAG-3'	5'-AAAAACCCACCCAAACAAACC-3'	57
		19q13.11	1st	5'-GTTAGTTGTTAGAGAGTTTTTTGG-3'	5'-CAACTCCCTAAACCCTCCTCC-3'	52
			2nd	5'-GTTTTTTGGTTAAGGTTATGGG-3'	5'-CTCCTACCACGAACCTACTCC-3'	54
		1p22.1	1st	5'-GGGTTTGAAGTTTTTTTTGTAGG-3'	5'-CAACAAAAACAATAAACAAAACC-3'	54
			2nd	5'-AAGATTATTTAGTTTTTTGGGG-3'	5'-CTTTACACCAATAAATTTAATACC-3'	50

### RT-PCR

Total RNA was isolated from PBMCs or lymph node cells using TRIzol Reagent (Invitrogen, Carlsbad, CA) and RT-PCR was performed using RNA LA PCR Kit (AMV) Ver. 1.1 (Takara Bio Inc., Otsu, Japan) according to the manufacturer's protocol. The *tax *and *GAPDH *gene transcripts were amplified using the following primers: RPX2 5'-CCGGCGCTGCTCTCATCCCGGT-3' and RPX5 5'-GGCCGAACATAGTCCCCCAGAG-3' (for *tax*), GAPDH1 5'-ATGGGGAAGGTGAAGGTCGGAGTC-3' and GAPDH1a 5'-CCATGCCAGTGAGCTTCCCGTTC-3' (for *GAPDH*) under following conditions: 2 minutes at 95°C for denaturation, 35 cycles of 30 sec at 95°C, 30 sec at 62°C, 30 sec at 72°C (for *tax*), 25 cycles of 30 sec at 95°C, 30 sec at 55°C, 30 sec at 72°C (for GAPDH) and 2 min at 72°C for final extension.

### Chromatin immunoprecipitation (ChIP) assay

ChIP assays were performed as described previously [40]. Briefly, ATL cell lines and fresh ATL cells from ATL patients (5 × l0^5 ^cells/antibody) were fixed with formaldehyde and then sonicated to obtain soluble chromatin. The chromatin solutions were immunoprecipitated with anti-acetyl-Histone H3 or anti-acetyl-Histone H4 (Upstate Biotechnology), or normal rabbit IgG, overnight at 4°C, and the immunoprecipitates were then collected with 50% protein A and G-Sepharose slurry preabsorbed with 0.1 mg/ml sonicated salmon sperm DNA. The resulting purified DNAs were subjected to PCR reactions using primer sets specific for 5'-LTR, *env*, pX and 3'-LTR. The sequences of the primers are shown in Table 1. To distinguish 5' and 3'-LTR, we used primers specific for *gag *and R region of LTR for amplification of 5'-LTR, and primers for pX region and U3 region were used for amplification of 3'-LTR. The PCR reactions were performed using FastStart Taq DNA Polymerase (Roche) with the following condition: 5 minutes at 95°C, 35 or 37 cycles of 30 sec at 95°C, 30 sec at each annealing temperature (Table 1), 30 sec at 72°C, and 2 min at 72°C. The PCR products were electrophoresed in an agarose gel and the results were analyzed using ATTO Densitograph Ver. 4.0. Values were calculated as the signal intensity of each sample normalized by that of the whole cell extract.

### Statistical analyses

Statistical analyses were performed using the Mann-Whitney's U-test and Student's t-test.

## Competing interests

The author(s) declare that they have no competing interests.

## Authors' contributions

YT conceived this project and carriers out most of experiments in Figs. 1, 2, 3, 5 and 6. KN established COBRA assay and performed experiments in Figs. 1 and 2. JY performed experiments in Fig. 7. MM established most of HTLV-I transformed cell lines, and analyzed experiments in Fig. 4. AO and NM provided sequential DNA samples from seroconverters, and analyzed the data. M.Matsuoka directed and supervised the experiments and interpretations All authors read and approved the final manuscript.
